# Case Report: *NEUROD1* c.-108G>C mutation in a ketosis-prone MODY6 patient: implications for genetic testing and DPP-4 inhibitor therapy

**DOI:** 10.3389/fendo.2025.1673765

**Published:** 2025-11-03

**Authors:** Zhuhai Shao, Lili Xu

**Affiliations:** Department of Endocrinology, The Affiliated Hospital of Qingdao University, Qingdao, China

**Keywords:** maturity-onset diabetes of the young, MODY6, *NEUROD1* gene, diabetic ketosis, DPP-4 inhibitor

## Abstract

This paper presents the diagnostic and therapeutic course of a 26-year-old male patient with maturity-onset diabetes of the young type 6 (MODY6) complicated by diabetic ketosis, resulting from a heterozygous *neurogenic differentiation factor 1* (*NEUROD1*) c.-108G>C mutation. The patient was admitted due to “dry mouth, polydipsia, and polyuria lasting for 2 months.” The diagnosis of MODY6 was established based on blood glucose levels, glycosylated hemoglobin results, familial co-segregation of the variant (maternally inherited), and genetic sequencing data. This study analyzes the similarities and discrepancies between this case and classic MODY6, highlights the diagnostic significance of genetic testing in atypical cases, and puts forward the indications for genetic testing in clinically suspected MODY cases. Following individualized therapy with saxagliptin and acarbose, the patient achieved stable blood glucose control without insulin after 6 months, with partial recovery of islet function. This case supports that *NEUROD1* mutations may retain incretin responsiveness, expanding therapeutic options for MODY6.

## 1 Introduction

Maturity-onset diabetes of the young (MODY) comprises a genetically heterogeneous group of monogenic forms of diabetes, characterized by autosomal dominant inheritance, early onset—typically before the age of 25—and primary β-cell dysfunction (1, 2). To date, 14 distinct MODY subtypes have been identified, each associated with specific genetic mutations and clinical phenotypes (3). Among these, MODY6, caused by heterozygous mutations in the *neurogenic differentiation factor 1* (*NEUROD1*) gene, is a rare subtype that exhibits considerable phenotypic variability, ranging from mild hyperglycemia to ketosis-prone diabetes (4, 5).

The *NEUROD1* gene, located on chromosome 2q32, encodes a basic helix-loop-helix (bHLH) transcription factor that plays a pivotal role in pancreatic β-cell development and the regulation of insulin secretion (6). Experimental studies have shown that *NEUROD1* modulates the expression of SUR1 (sulfonylurea receptor 1), a critical subunit of β-cell ATP-sensitive K^+^ (K_ATP_) channels (composed of SUR1 and Kir6.2). The SUR1-Kir6.2 complex mediates glucose-dependent insulin secretion (GSIS): increased glucose metabolism raises intracellular ATP levels, closing K_ATP_ channels and triggering Ca^2+^ influx to promote insulin release. Thus, *NEUROD1* dysfunction links to impaired GSIS by downregulating SUR1 expression (6, 7). While most reported *NEUROD1* mutations cluster within the transactivation domain and are typically associated with classic MODY6 phenotypes—characterized by non-ketotic, insulin-independent diabetes (4)—emerging evidence indicates that variants in the 5′ untranslated region (5′UTR) may disrupt transcriptional regulation, potentially resulting in atypical clinical presentations, including diabetic ketosis (8, 9).

Specifically, Su et al. (9) demonstrated that dinucleotide composition in the UTR region is a major determinant of RNA stability; variants altering this composition can reduce mRNA half-life and subsequent protein expression, which may explain why 5’UTR variants of *NEUROD1* differ from transactivation domain mutations in phenotypic severity. Additionally, Horikawa et al. (5) reported that Japanese patients with *NEUROD1*-deficient MODY6 already exhibited heterogeneous β-cell dysfunction, with some cases showing near-normal insulin secretion and others presenting with severe impairment—hinting that *NEUROD1* variant location (e.g., coding vs. non-coding regions) contributes to phenotypic variability. In our case, the *NEUROD1* c.-108G>C variant in the 5’UTR may further expand this heterogeneity by disrupting transcriptional initiation or mRNA stability, ultimately leading to ketosis—a phenotype rarely reported in classic MODY6.

Building on the aforementioned evidence that 5’UTR variants of *NEUROD1* may drive atypical MODY6 phenotypes, our study provides the first clinical evidence linking the *NEUROD1* c.-108G>C 5’UTR variant to ketosis-prone MODY6—a presentation that challenges the traditional view of MODY6 as non-ketotic. Moreover, the patient’s positive response to DPP-4i therapy offers new insights into therapeutic options for *NEUROD1*-associated MODY6, which has previously been considered primarily insulin dependent. Here, we report a novel association between the *NEUROD1* c.-108G>C variant and ketosis-prone MODY6, characterized by a favorable therapeutic response to dipeptidyl peptidase-4 inhibitor (DPP-4i) therapy. These findings highlight the importance of expanding genetic testing criteria to encompass atypical presentations of diabetes.

## 2 Case presentation

### 2.1 Clinical history

**Figure 1:**
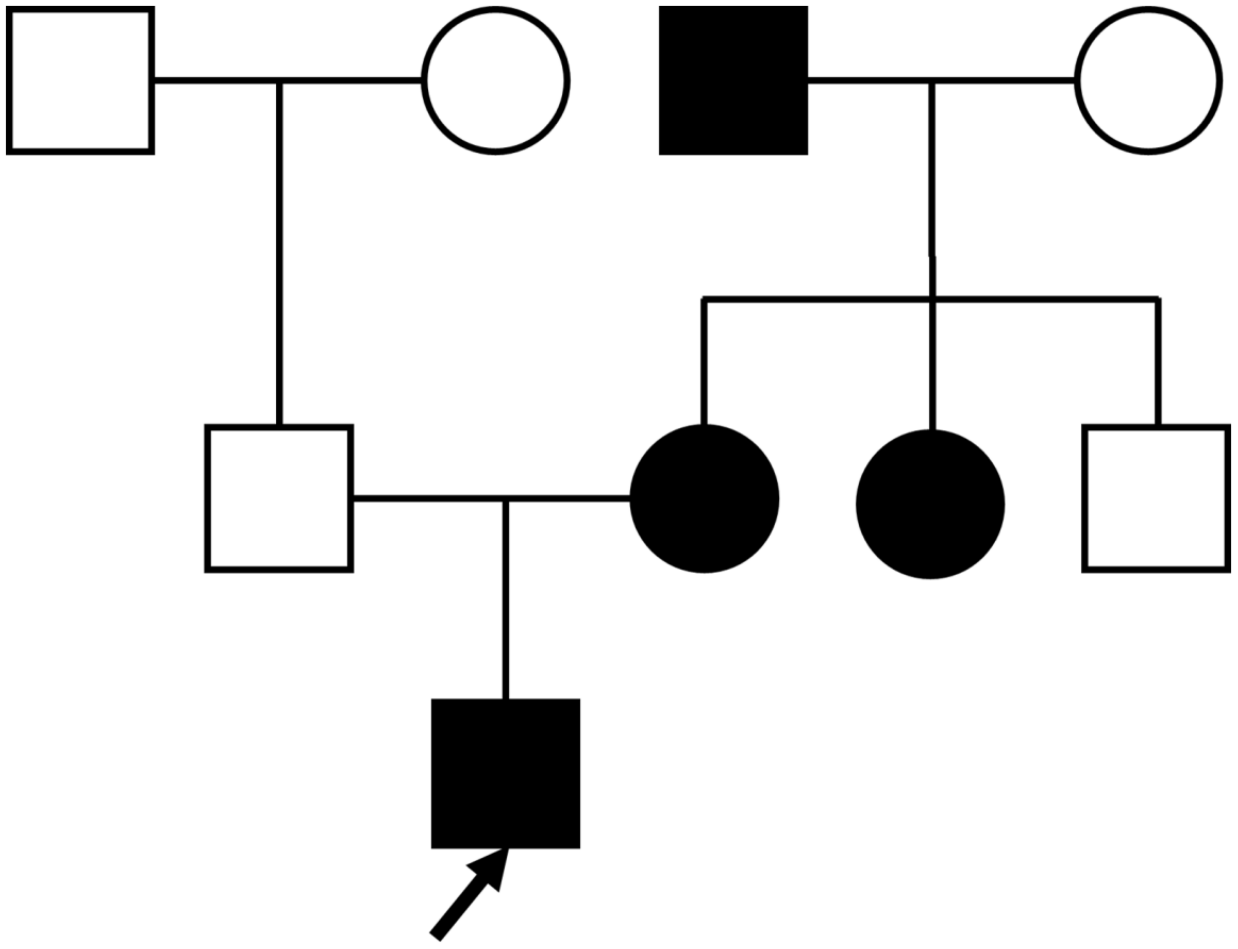
Proband’s family tree: Squares represent males, and circles represent females. The black symbol represents individuals with diabetes; the blank symbol represents normal individuals. The arrow indicates the proband.

A 26-year-old unmarried male was admitted to the hospital with complaints of dry mouth, polydipsia, and polyuria persisting for two months. These symptoms began following a febrile episode two months prior to admission and were accompanied by foamy urine and an unintentional weight loss of 7kg. Outpatient evaluation revealed a fasting blood glucose level of 11.84 mmol/L. Regarding family history, his father was non-diabetic, whereas his mother, maternal aunt, and maternal grandfather were all diagnosed with diabetes, suggesting an autosomal dominant pattern of inheritance (**Figure 1**).

### 2.2 Physical examination

On admission, the patient was afebrile (36.5°C) with a respiratory rate of 20 breaths per minute, a pulse of 111 beats per minute, and a blood pressure of 145/87 mmHg. Anthropometric assessment revealed a height of 186 cm, weight of 87.50 kg, body mass index (BMI) of 25.30 kg/m², waist circumference of 88 cm, and hip circumference of 106 cm. A comprehensive physical examination, including evaluation of the cardiovascular, respiratory, abdominal, and neurological systems, revealed no significant abnormalities.

### 2.3 Laboratory findings

The patient’s glycated hemoglobin (HbA1c) level was elevated at 12.80%. An insulin release test revealed a fasting insulin level of 0.98 μIU/ml (reference range: 2.60–24.90 μIU/ml), with 1h and 2h postprandial insulin levels of 3.90 μIU/ml and 6.82 μIU/ml, respectively. A C-peptide release test showed markedly reduced fasting C-peptide at 0.28 ng/ml (reference range: 1.10–4.40 ng/ml), with postprandial 1h and 2h C-peptide levels of 0.67 ng/ml and 0.92 ng/ml, respectively. Testing for islet autoantibodies, including glutamic acid decarboxylase autoantibodies, was negative. Blood lipid profile results were within normal limits: total cholesterol (TC) 4.63 mmol/L (reference value: 2.90–5.68 mmol/L), triglycerides (TG) 0.55 mmol/L (reference value: 0.00–1.70 mmol/L), low-density lipoprotein cholesterol (LDL-C) 3.00 mmol/L (reference value: 0.00–3.37 mmol/L), and high-density lipoprotein cholesterol (HDL-C) 0.95 mmol/L (reference value: 0.91–2.06 mmol/L). Liver function indicators were normal: alanine aminotransferase (ALT) 28 U/L (reference value: 9–50 U/L) and aspartate aminotransferase (AST) 25 U/L (reference value: 15–40 U/L). Renal function was also normal: serum creatinine (Scr) 53 μmol/L (reference value: 57–97 μmol/L), uric acid (UA) 257 μmol/L (reference value: 293–430 μmol/L), and blood urea nitrogen (BUN) 2.34 mmol/L (reference value: 3.10–8.00 mmol/L). The urinary albumin-to-creatinine ratio (UACR) was 4.61 mg/g. Urinalysis showed 3+ ketone bodies and 4+ glucose. Arterial blood gas (ABG) analysis revealed no metabolic acidosis, with pH 7.43 (reference value: 7.35–7.45), partial pressure of arterial oxygen (PaO_2_) 87 mmHg (reference value: 80–100 mmHg), partial pressure of arterial carbon dioxide (PCO_2_) 40 mmHg (reference value: 35–45 mmHg), standard bicarbonate (SB) 26.4 mmol/L (reference value: 22–27 mmol/L), and base excess (BE) 2.2 mmol/L (reference value: −3.0–3.0 mmol/L).

### 2.4 Genetic analysis

**Figure 2:**
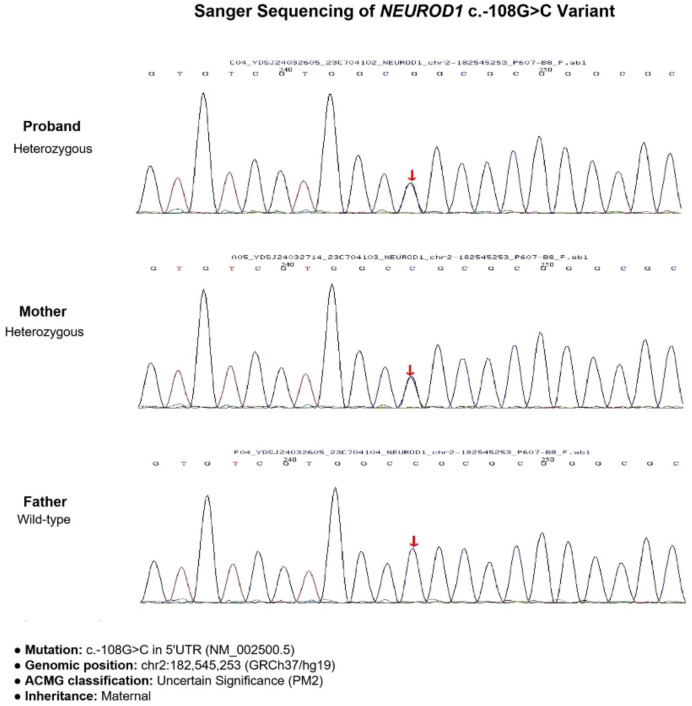
Gene sequencing results of the proband and his parents. The proband and his mother both carried the heterozygous variant.

Whole-exome sequencing identified a heterozygous variant in the *NEUROD1* gene (NM_002500.5): c.-108G>C, located in the 5′UTR (**Figure 2**). According to American College of Medical Genetics and Genomics (ACMG) guidelines (10), this variant was classified as of “uncertain significance” (PM2_Supporting: absent from population databases; PP1_Moderate: co-segregation with diabetes within the maternal lineage). Sanger sequencing confirmed the presence of the variant in both the proband and his diabetic mother, while it was absent in his non-diabetic father. No pathogenic variants were identified in other MODY-associated genes, including *GCK* and *HNF1A*.

### 2.5 Diagnosis and treatment

Based on the clinical presentation, autosomal dominant family history, and genetic findings, a diagnosis of MODY6 was established. After admission, the patient received intravenous fluid replacement and continuous subcutaneous insulin pump therapy with insulin lispro injection. The insulin regimen was set as follows: basal rate 16.7 U/day, with 5 U before breakfast, 4 U before lunch, and 5 U before dinner, totaling 30.7 U/day. The dosage was dynamically adjusted according to capillary blood glucose monitoring results (6–8 measurements daily). Concomitant medication included saxagliptin 5 mg once daily and acarbose 50 mg three times daily (to be chewed with meals). After urinary ketone bodies turned negative, the patient was discharged. One month post-discharge, partial recovery of islet function was observed, with a fasting C-peptide level of 0.58 ng/ml. At three months, fasting C-peptide had increased to 1.16 ng/ml, and HbA1c had decreased to 6.2%. By 6 months, insulin therapy was discontinued, and glycemic control remained stable without recurrence of ketosis. The detailed changes in the patient’s clinical indicators and treatment adjustments during the entire course are summarized in **Table 1**.

**Table 1:** Clinical indicators and treatment regimens of the patient at baseline and follow-up (1, 3, and 6 months).

Specific indicator	Baseline	1-month follow-up	3-month follow-up	6-month follow-up
General condition	–	–	–	–
Age (years)	26	–	–	–
Height (cm)	186	–	–	–
Weight (kg)	87.50	–	–	–
BMI (kg/m²)	25.30	–	–	–
Waist circumference (cm)	88	–	–	–
Hip circumference (cm)	106	–	–	–
Lipid indicators	–	–	–	–
TG (mmol/l)	0.55	–	–	–
TC (mmol/l)	4.63	–	–	–
LDL-C (mmol/l)	3.00	–	–	–
HDL-C (mmol/l)	0.95	–	–	–
Glucose-related indicators	–	–	–	–
FBG (mmol/L)	11.84	3.55	5.04	5.20
HbA1c (%)	12.80	–	6.20	–
Fasting insulin (μIU/ml)	0.98	2.62	–	–
Fasting C-peptide (ng/ml)	0.28	0.58	1.16	–
Liver/kidney function indicators	–	–	–	–
ALT (U/L)	28	–	–	–
AST (U/L)	25	–	–	–
UA (μmol/L)	257	–	–	–
BUN (mmol/L)	2.34	–	–	–
Scr (μmol/L)	53	–	–	–
Urine indicators	–	–	–	–
UACR (mg/g)	4.61	–	–	–
Urinary ketone bodies	3+	–	–	–
Urinary glucose	4+	–	–	–
ABG indicators	–	–	–	–
pH value	7.43	–	–	–
PaO_2_ (mmHg)	87	–	–	–
PaCO_2_ (mmHg)	40	–	–	–
SB (mmol/L)	26.4	–	–	–
BE (mmol/L)	2.2	–	–	–
Treatment regimen	–	–	–	–
Insulin pump (U/day)	30.7 (Basal 16.7 + Preprandial 14)	26.8 (Basal 15.3 + Preprandial 11.5)	24.0 (Basal 12.5 + Preprandial 11.5)	–
Saxagliptin (mg/day)	5	5	5	5
Acarbose (mg/dose × doses/day)	5 × 3	5 × 3	5 × 3	5 × 3

BMI, body mass index; TG, triglycerides; TC, total cholesterol; LDL-C, low-density lipoprotein cholesterol; HDL-C, high-density lipoprotein cholesterol; FBG, fasting blood glucose; HbA1c, glycated hemoglobin; ALT, alanine aminotransferase; AST, aspartate aminotransferase; UA, uric acid; BUN, blood urea nitrogen; Scr, serum creatinine; UACR, urinary albumin-to-creatinine ratio; ABG, arterial blood gas; PaO_2_, partial pressure of arterial oxygen; PaCO_2_, partial pressure of arterial carbon dioxide; SB, standard bicarbonate; BE, base excess.

## 3 Materials and methods

### 3.1 Study subjects

This study included one 26-year-old male patient diagnosed with MODY6 and his family members (father and mother). The patient was admitted to the Department of Endocrinology, Affiliated Hospital of Qingdao University, due to typical diabetic symptoms combined with a family history of diabetes. All participants provided written informed consent, and the study was approved by the Medical Ethics Committee of the Affiliated Hospital of Qingdao University (Ethical Approval No. QYFYWZLL30623).

### 3.2 Laboratory tests

Fasting blood glucose (FBG) was measured using the glucose oxidase method. HbA1c was determined via high-performance liquid chromatography. Serum samples (3 ml venous blood, centrifuged at 3000 rpm for 10min for separation) were used to detect insulin and C-peptide levels by chemiluminescent immunoassay. Glutamic acid decarboxylase autoantibodies were tested using enzyme-linked immunosorbent assay. Blood lipid indicators, liver function, and renal function were measured on an automatic biochemical analyzer with corresponding commercial kits. Urinalysis was performed using a urine chemistry analyzer to detect ketone bodies and glucose, and ABG analysis was conducted with a blood gas analyzer.

### 3.3 Genetic analysis

Peripheral venous blood (2 ml) was collected from the proband and his father and mother. Whole-exome sequencing (WES) was performed by MyGenostics (Beijing) Co., Ltd., with an average sequencing depth of 265.33× and a target region coverage of 99.79%. Sanger sequencing was used for family co-segregation verification of the identified variant. Variant pathogenicity was classified according to the ACMG guidelines.

## 4 Discussion

This study describes a patient with MODY6 presenting with diabetic ketosis, carrying a heterozygous *NEUROD1* c.-108G>C variant. Although classified as “Uncertain Significance” by ACMG criteria (10, 11) (PM2_Supporting: absent in population databases; PP1_Moderate: maternal cosegregation), multiple lines of evidence support its pathogenicity. The proband exhibited classic MODY6 features (early-onset, non-autoimmune diabetes, β-cell dysfunction) along with atypical ketosis, consistent with previous reports associating *NEUROD1* transactivation domain mutations with ketotic episodes (4, 5). Pedigree analysis confirmed the mutation was inherited from the diabetic mother while the non-diabetic father was wild-type (**Figure 2**), following an autosomal dominant pattern (3). Particularly, the c.-108G>C variant is located in the 5’UTR region, which contains key elements for transcriptional regulation, potentially affecting the expression regulation of *NEUROD1* (12).

While functional studies are required to fully elucidate the molecular consequences, the observed clinical correlation in this family provides strong evidence supporting the disease association. These findings diverge from the classic MODY6 phenotype—characterized by the absence of ketosis and insulin independence for over five years after diagnosis—thereby underscoring the phenotypic heterogeneity of *NEUROD1* mutations (3, 13). Based on the genotype-phenotype correlation and familial evidence, we propose re-evaluating the ACMG classification (PM1+PM2+PP1+PP4) to “Likely Pathogenic” for similar cases with strong phenotypic support.

### 4.1 Rationale for individualized treatment selection

The therapeutic strategy for this patient was formulated based on the integration of genetic background, clinical manifestations, and pharmacodynamic evidence, reflecting the principle of precision medicine in MODY management.

Selection of saxagliptin as the preferred DPP-4 inhibitor was supported by three key considerations. First, pharmacokinetic data indicate that saxagliptin has a favorable safety profile in Asian populations with minimal drug-drug interactions, which is critical for long-term maintenance therapy (14). The patient, a 26-year-old Chinese male with normal baseline liver and renal function, required a treatment option with low long-term organ toxicity, and saxagliptin’s predominantly renal excretion (with no need for dose adjustment in normal renal function) met this requirement. Second, the mechanism of DPP-4i, which involves upregulating anti-apoptotic factors to enhance β-cell survival and prolonging the half-life of endogenous incretins (e.g., GLP-1 and GIP) to stimulate insulin secretion, is highly congruent with the core objective of preserving residual β-cell function in *NEUROD1*-deficient MODY6 (15, 16). Given that *NEUROD1* mutations impair β-cell development and insulin secretion rather than causing irreversible β-cell destruction, promoting β-cell survival is particularly relevant for this patient. Third, saxagliptin displays greater *in vitro* DPP-4 inhibitory potency than some other DPP-4 inhibitors (e.g., sitagliptin), rendering it more suitable for patients with severe baseline β-cell dysfunction, as illustrated by our patient (fasting C-peptide 0.28 ng/ml) (17).

Adjunctive use of acarbose was justified by the patient’s prominent postprandial hyperglycemia and the need for rapid glycemic control during ketosis resolution. Acarbose acts locally in the gastrointestinal tract to delay carbohydrate absorption (by inhibiting α-glucosidase) without systemic absorption, avoiding potential interference with saxagliptin’s mechanism of action (prolonging incretin half-life via DPP-4 inhibition). This combination addressed two key clinical needs simultaneously: acarbose rapidly reduced postprandial glucose spikes to prevent further ketosis exacerbation, while saxagliptin targeted the underlying β-cell dysfunction.

### 4.2 Clinical implications for MODY6 management

Current clinical guidelines for monogenic diabetes typically suggest insulin as the primary treatment for MODY6, based chiefly on observational data from cases with *NEUROD1* coding region mutations (18, 19). However, this case demonstrates that a “short-term intensive insulin + long-term DPP-4 inhibitor” regimen can achieve insulin independence, with stable glycemic control and partial β-cell recovery. We propose that, for MODY6 patients with *NEUROD1* variants (especially 5’UTR variants) and ketosis (without severe metabolic acidosis), DPP-4 inhibitors may be considered as maintenance therapy after short-term insulin to correct ketosis.

This case carries important implications for refining the indications for MODY genetic testing. Genetic evaluation should be considered in patients with antibody-negative diabetes who exhibit any of the following features (20): (1) unexplained ketosis, (2) early onset diabetes (even beyond age 25) accompanied by a family history suggestive of MODY, and (3) clinical features indicative of monogenic diabetes, even in the presence of variants classified as “uncertain significance” by ACMG criteria. Notably, the patient’s mother, who carried the same *NEUROD1* mutation, exhibited a milder diabetic phenotype, suggesting variable expressivity. This highlights the importance of comprehensive genotypic and phenotypic evaluation of family members when assessing potential monogenic diabetes.

### 4.3 Study limitations

The limitations of this study include (1) the lack of functional validation and bioinformatics analysis (e.g., luciferase reporter assay) for the *NEUROD1* c.-108G>C variant, which would help confirm its impact on transcriptional activity, and (2) short-term follow-up (6 months), which cannot confirm the long-term durability of glycemic control and β-cell recovery—future work will extend follow-up to 12 and 24 months to monitor for potential relapse of ketosis or β-cell function decline. Future research should incorporate *in vitro* experiments to assess the impact of the c.-108G>C variant on *NEUROD1* transcriptional activity, as well as expand sample sizes to evaluate the long-term efficacy of DPP-4i in patients with *NEUROD1*-MODY. Furthermore, testing additional family members would enhance the robustness of the genotype-phenotype correlation analysis.

## Data Availability

The original contributions presented in the study are included in the article/supplementary material. Further inquiries can be directed to the corresponding author.
